# *In-silico* identification of deleterious non-synonymous SNPs of *TBX1* gene: Functional and structural impact towards 22q11.2DS

**DOI:** 10.1371/journal.pone.0298092

**Published:** 2024-06-21

**Authors:** Maitha Almakhari, Yan Chen, Amanda Shen-Yee Kong, Danesh Moradigaravand, Kok-Song Lai, Swee-Hua Erin Lim, Jiun-Yan Loh, Sathiya Maran

**Affiliations:** 1 Oxford Nanopore Department, Omics Centre of Excellence (Biogenix Labs) G42 Healthecare, Abu Dhabi, United Arab Emirates; 2 Hainan Key Laboratory for Conservation and Utilization of Tropical Marine Fishery Resources & Key Laboratory of Utilization and Conservation for Tropical Marine Bioresources of Ministry of Education, Hainan, PR China; 3 School of Pharmacy, Monash University Malaysia, Jalan Lagoon Selatan, Bandar Sunway, Selangor Darul Ehsan, Malaysia; 4 Laboratory for Infectious Disease Epidemiology, KAUST Smart-Health Initiative and Biological and Environmental Science and Engineering (BESE) Division, King Abdullah University of Science and Technology (KAUST), Thuwal, Makkah, Saudi Arabia; 5 KAUST Computational Bioscience Research Center (CBRC), King Abdullah University of Science and Technology (KAUST), Thuwal, Makkah, Saudi Arabia; 6 Health Sciences Division, Abu Dhabi Women’s College, Higher Colleges of Technology, Abu Dhabi, United Arab Emirates; 7 Faculty of Applied Sciences, UCSI University, Kuala Lumpur, Malaysia; 8 Tropical Futures Institute, James Cook University Singapore, Singapore, Singapore; CSIR-IHBT: Institute of Himalayan Bioresource Technology CSIR, INDIA

## Abstract

The *TBX1* gene plays a critical role in the development of 22q11.2 deletion syndrome (22q11.2DS), a complex genetic disorder associated with various phenotypic manifestations. In this study, we performed in-silico analysis to identify potentially deleterious non-synonymous single nucleotide polymorphisms (nsSNPs) within the *TBX1* gene and evaluate their functional and structural impact on 22q11.2DS. A comprehensive analysis pipeline involving multiple computational tools was employed to predict the pathogenicity of nsSNPs. This study assessed protein stability and explored potential alterations in protein-protein interactions. The results revealed the rs751339103(C>A), rs780800634(G>A), rs1936727304(T>C), rs1223320618(G>A), rs1248532217(T>C), rs1294927055 (C>T), rs1331240435 (A>G, rs1601289406 (A>C), rs1936726164 (G>A), and rs911796187(G>A) with a high-risk potential for affecting protein function and stability. These nsSNPs were further analyzed for their impact on post-translational modifications and structural characteristics, indicating their potential disruption of molecular pathways associated with *TBX1* and its interacting partners. These findings provide a foundation for further experimental studies and elucidation of potential therapeutic targets and personalized treatment approaches for individuals *affected* by 22q11.2DS.

## Introduction

The 22q11.2 deletion syndrome is the most common deletion syndrome caused by deletion at locus q11.2 on Chromosome 22 [1]. Studies have indicated that it occurs in one of every 4000 live births [2]. Clinical characteristics of 22q11.2 DS vary greatly in terms of penetrance and expressivity [3]. This deletion is also reported to causes a multisystem illness due to haploinsufficiency of about 50 genes [4]. Commonly reported are; congenital heart defects, thymic and parathyroid hypoplasia and aplasia, craniofacial dysmorphisms, cleft palate, and learning difficulties are distinguishing characteristics [5–7].

Clinical variations among families with 22q11.2 DS are extremely diverse [8]. The high clinical variability is a major concern as it can result in delays in diagnosis, particularly for patients without typical symptoms such as conotruncal anomalies and palatal abnormalities associated with congenital heart disease [4, 9, 10]. Lee and colleagues [11] reported that the syndrome’s low penetrance and high specificity further exacerbate the uncertainty surrounding incomplete penetrance, with the extent of clinical expression differing not only between families but also within affected individuals of the same family. Consequently, clinicians face difficulties in predicting the severity of the syndrome in foetuses, even when parents present with mild or normal phenotypes. Timely diagnosis is critical since it leads to better clinical outcomes, early protocolled care, including rehabilitative and modified educational programmes, a suitable vaccine schedule, and proper genetic counselling for the patient and their caregivers [8].

Although multiple genes have been associated with 22q11.2DS, *TBX1* (T-box1) is considered a key contributor to this disorder [12]. This is primarily due to the discovery of heterozygous *TBX1* mutations in numerous patients with the 22q11.2DS phenotype [13–18]. Furthermore, in-vivo studies have provided further evidence to support *TBX1* as the causative gene for 22q11.2DS [14].

Single nucleotide polymorphisms (SNPs) accounts of 90% of genetic variations in humans, and nonsynonymous SNPs (nsSNPs) are of interest as amino acids change, potentially impacting the structure and function of proteins, resulting in deleterious effects [19]. The vast number of nsSNPs in the human genome necessitates an extensive investigation to understand their significance and potential associations with disease susceptibility [20]. *In silico* analysis employing streamlined bioinformatic tools has proven to be effective in investigating a large number of variants before experimental testing in vitro or in vivo assays [21–24]. These computational tools and databases offer a cost and labour-effective potential in exploring their implications for human health and disease.

Understanding the role of *TBX1* is crucial as it provides insights into the molecular mechanisms underlying the syndrome’s diverse symptoms. Elucidating the pathogenesis of nsSNPs of TBX1 influences the identification of potential therapeutic targets and diagnostic markers for this complex condition. In-silico analysis, using computational tools and algorithms, becomes essential due to the complexity and scale of genetic data involved in 22q11.2 deletion syndrome. This method enables simulation and analysis of biological processes, predicting protein structures, and understanding gene interactions without expensive and time-consuming laboratory experiments [25–28]. Previous studies have demonstrated the feasibility of in-silico analysis in identifying potential interactions of genes, pathways, or cellular mechanisms, offering valuable clues for further experimental validation [21, 26, 28–34]. Hence, the objective of this study is to utilize in-silico analysis to investigate the role of the TBX1 gene within the context of 22q11.2 deletion syndrome. This aims to enhance understanding of the syndrome’s molecular basis and its broad array of clinical manifestations.

## Results

### Predicting Deleterious nsSNPs of *TBX1* gene

**Fig 1:**
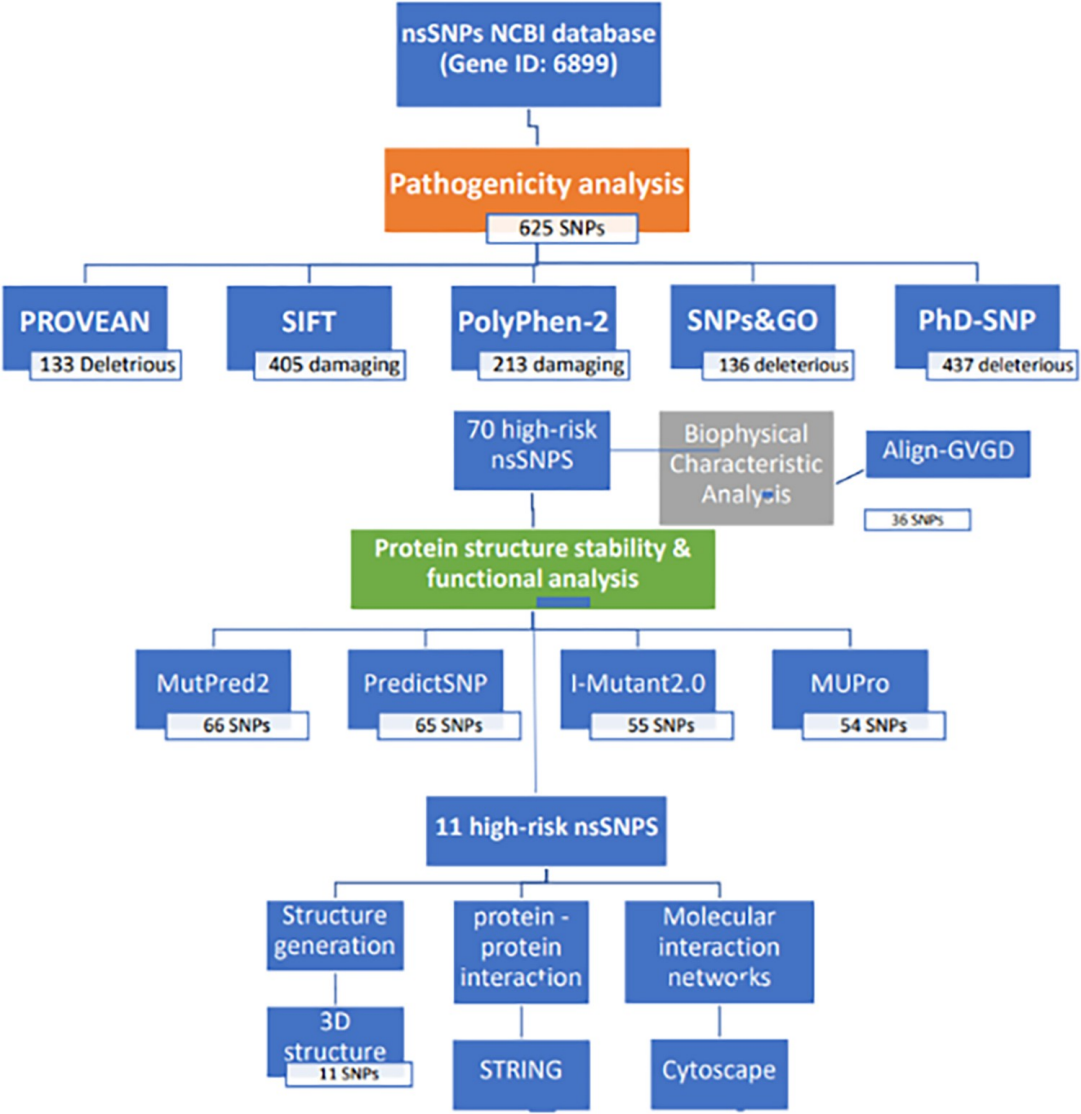
Diagrammatic flow chart of bioinformatic tools used and nsSNPs analysed.

A total of 625 nsSNPs of *TBX1* were retrieved from the NCBI. Further analysis with PROVEAN, SIFT, PolyPhen-2, SNPs&GO, and PhD-SNP was carried out to identify highly pathogenic nsSNPs. PROVEAN predicted 133 nsSNPs as pathogenic and 530 as neutral. SIFT predicted 405 nsSNPs as damaging and 220 variants as tolerated. PolyPhen-2 predicted 244 nsSNPs as benign, 168 nsSNPs as possibly damaging, and 213 variants as probably damaging. SNPs&GO predicted 136 variants to cause disease while 489 variants as neutral, whereas PhD-SNP predicted 437 nsSNPs to cause disease and 188 variants as neutral. Overview of the whole methodology and the nsSNPs analysed summarised in a schematic diagram (Fig 1).

To determine the most highly damaging nsSNPs, all the tools were considered, and only variants that were identified as pathogenic by all the tools were selected. As a result, 70 nsSNPs were predicted as pathogenic and were further analyzed (S1 Table in S1 File).

### Prediction of high-risk nsSNPs on protein stability

Protein structure stability analysis identified 24 variants with a score of 0.9, indicating high pathogenic potential. Among these variants, rs1936727304 (T>C) had the highest score of 0.973 and was predicted to cause an Altered Ordered interface with a 0.3 probability and an Altered Metal-binding with a 0.27 probability. Similarly, rs911796187 had a score of 0.956 and was predicted to cause Loss of Allosteric site at R296 with a 0.37 probability and an Altered Disordered interface with a 0.3 probability. Another variant, rs1331240435 (A>G), had a score of 0.949 and was predicted to cause Altered Metal binding with a probability of 0.29 and Loss of Allosteric site at D161 with a 0.27 probability. Additionally, rs1601289406 had a score of 0.947 and was predicted to cause Altered Metal binding with a probability of 0.65 and an Altered Ordered interface with a 0.26 probability. Further analysis using PredictSNP, I-Mutant2.0, and MuPro tools identified 11 nsSNPs with high-risk potential, which can reduce protein stability (Table 1)

**Table 1:** High-risk nsSNPs of *TBX1* predicted using MutPred2, PredictSNP, I-Mutant 2.0 and MuPro.

		MutPred2		PredictSNP		I-Mutant2.0		MuPro	
SNP ID	Mutation	Score	Pred	Pred	Score	Pred	Score	Pred	Score
rs751339103	R296W	0.973	pathogenic	deleterious	87%	decrease	6	decrease	-1
rs780800634	R280P	0.956	pathogenic	deleterious	87%	decrease	6	decrease	-0.42
rs1936727304	L159P	0.949	pathogenic	deleterious	87%	decrease	5	decrease	-0.97
rs911796187	R296P	0.947	pathogenic	deleterious	87%	decrease	1	decrease	-0.56
rs1223320618	G183E	0.935	pathogenic	deleterious	87%	decrease	8	decrease	-0.05
rs1248532217	F295S	0.917	pathogenic	deleterious	87%	decrease	6	decrease	-0.21
rs1294927055	R169C	0.915	pathogenic	deleterious	87%	decrease	5	decrease	-0.54
rs1331240435	Y156C	0.913	pathogenic	deleterious	87%	decrease	6	decrease	-0.71
rs1601289406	H196P	0.912	pathogenic	deleterious	87%	decrease	7	decrease	-1
rs1936726164	G149D	0.907	pathogenic	deleterious	87%	decrease	4	decrease	-0.41
rs1936783557	R240C	0.904	pathogenic	deleterious	87%	decrease	2	decrease	-1

The effect of the 11 nsSNPs, which were predicted to have high-risk potential, was assessed using the MUPred post-translational modification analysis. The probability scores above 0.5 were considered "damaging," while scores over 0.75 were considered "harmful" nsSNPs. The analysis revealed that all 11 nsSNPs altered post-translational modifications and structural characteristics with scores >0.90. Specifically, the amino acid substitutions R296P and Y156C were predicted to cause the loss of allosteric sites, while L159P and H196P were predicted to alter metal-binding sites. These results suggest that the identified nsSNPs can significantly affect the protein’s stability and function, and further research is needed to explore their potential role in disease development.

### Biophysical characteristic analysis

Align-GVGD analysis of the 70 *TBX1* nsSNPs were categorized into seven classes: C65 (n = 36), C55 (n = 10), C45 (n = 3), C35 (n = 3), C25 (n = 4), C15 (n = 12), and C0 (n = 2). The middle class, C35, along with classes C0, C15, and C25, denote mutations with no apparent effect on protein function, while classes C45, C55, and C65 indicate mutations with functional impact on the protein. In this study, Align-GVGD analysis identified that all 11 high-risk *TBX1* nsSNPs fall within the C65 class. These nsSNPs include rs751339103 (C>A), rs780800634 (G>A), rs1936727304 (T>C), rs1223320618(G>A), rs1248532217(T>C), rs1294927055 (C>T), rs1331240435, rs1601289406, rs1936726164, rs911796187, and rs1331240435 (A>G).

### Protein-protein and molecular pathway interaction

**Fig 2:**
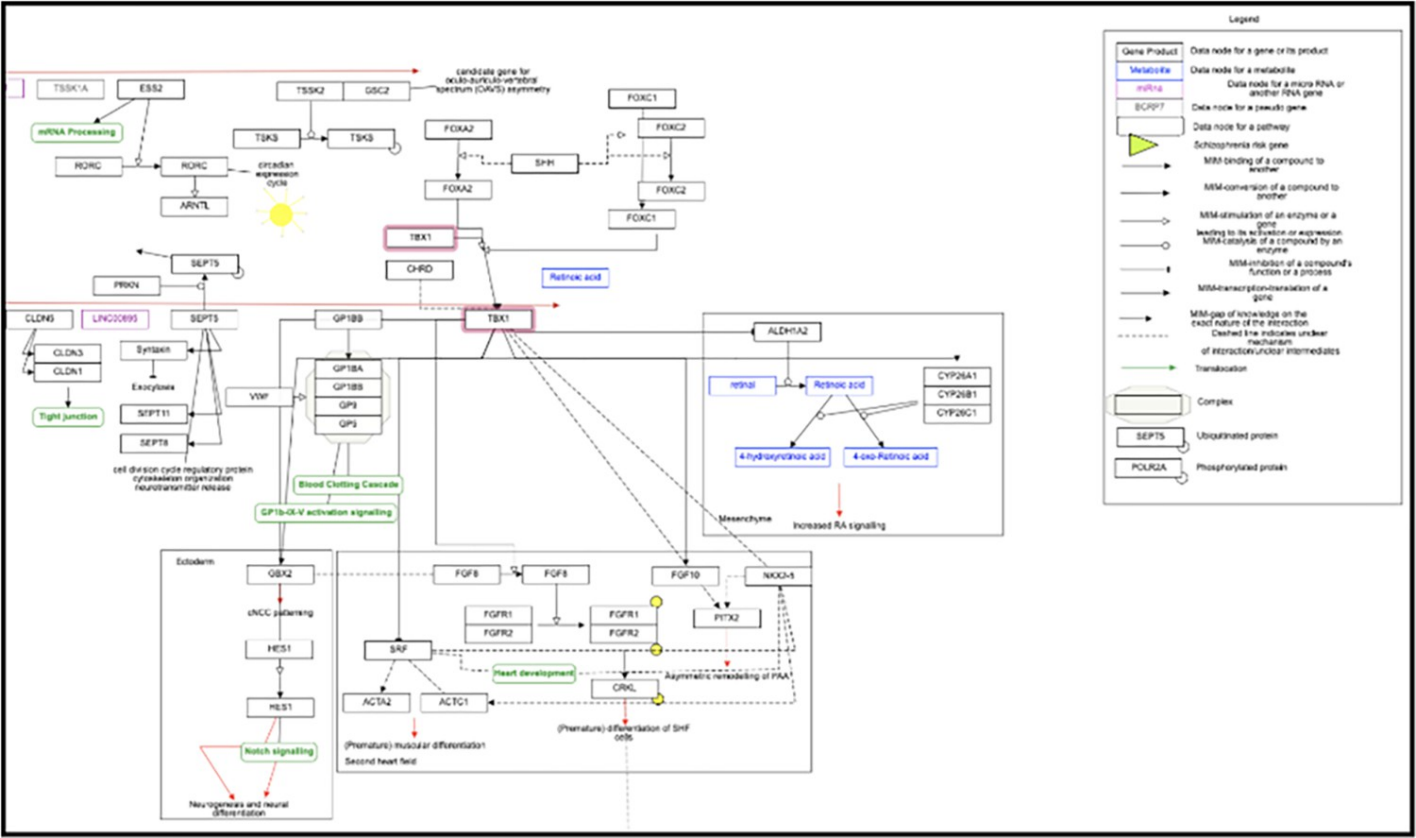
Network of molecular interactions and biological processes for the *TBX1* gene.

The functional impact of *TBX1* and its interacting partners was explored through protein-protein interaction (PPI) analysis in a study by Mondal et al. (2022) [35]. The analysis revealed that *TBX1* showed a high confidence score for interaction with *SEPT5*, *NKX2-5*, *BANF1*, *SLC25A1*, *GNB1L*, *UCP1*, *PRDM16*, *TMEM26*, *CIDEA*, and *DROSHA*. The study also used molecular interaction network and biological pathway visualization to show that *TBX1* is linked to the FOX gene family, specifically the *FOXC2* gene (MIM 602402) and *FOXC1* gene (MIM 601090) (Fig 2).

### Predict structural Alteration using SWISS-Model

**Fig 3:**
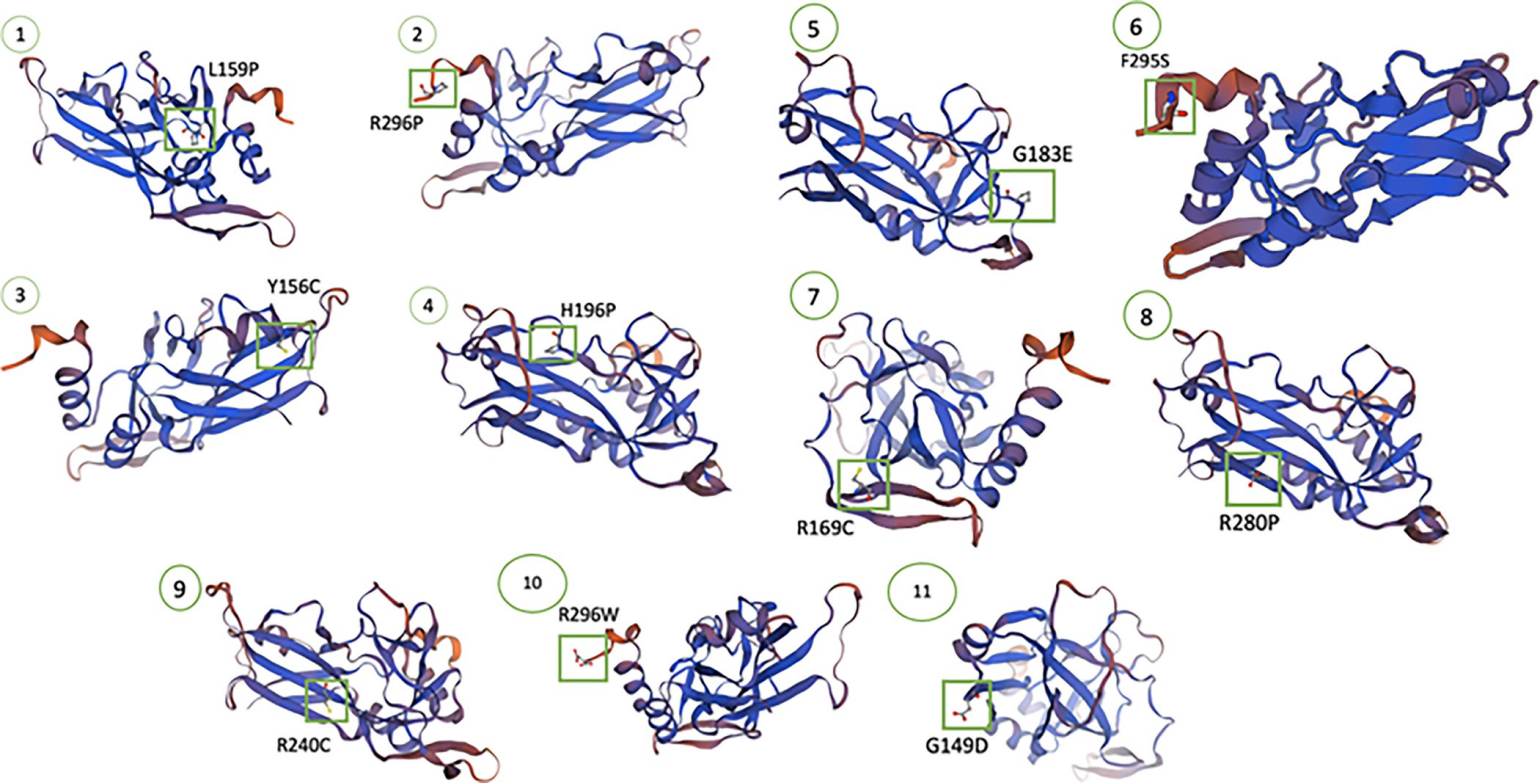
Show the 3D protein structure of 11 highly pathogenic nsSNPs. (1) Leucine to Proline at position 159, (2) Arginine to Proline at position 296, (3) Tyrosine to Cysteine at position 156, (4) Histidine to Proline at position 196, (5) Glycine to Glutamic Acid at position 183, (6) Phenylalanine to Serine at position 295, (7) Arginine to Cysteine at position 169, (8) Arginine to Proline at position 280, (9) Arginine to Cysteine at position 240, (10) Arginine to Tryptophan at position 296, and (11) Glycine to Aspartic Acid at position 149.

The 3D protein structure of these 11 nsSNPs were generated using SWISS-MODEL (Fig 3 and S2 Fig in S1 File). These structures help in understanding the impact of the mutations on the *TBX1* protein structure and its function

### Protein docking using pyDockWEB and ClusPro2.0

The results from protein docking simulations between *TBX1* and *FGF8*, and between *TBX1* and *BMPER* (Table 2) are shown. The tables present the binding energies for different mutants and their respective center and lowest energy scores. Docking of wild-type TBX1 with FGF8 resulted in a center energy of -754.6 kcal/mol and a lowest energy of -882.4 kcal/mol. Overall, the docking results with FGF8 for all *TBX1* mutant proteins exhibited lower energy values compared to the wild-type, except for the R280P mutation, which had a higher lowest energy of -861.5 kcal/mol. Conversely, docking of wild-type *TBX1* with BMPER yielded a center energy of -1243.2 kcal/mol and a lowest energy of -1362.2 kcal/mol. Overall, the docking results with BMPER for all TBX1 mutant proteins showed lower energy values compared to the wild-type, except for the L159P and R296W mutations, which had higher center energy values of -1176.4 kcal/mol and -1205.4 kcal/mol, respectively. These findings provide insights into the binding affinity between *TBX1* and FGF8/BMPER where low energy scores indicate a stronger interaction.

**Table 2:** Protein docking *TBX1* with *FGF8* and *BMPER*.

with FGF8	Center (kcal/mol)	Lowest Energy (kcal/mol)	with BMPER	Center (kcal/mol)	Lowest Energy (kcal/mol)
WT	-754.6	-882.4	WT	-1243.2	-1362.2
G149D	-827.9	-983.3	G149D	-1417.3	-1622.2
Y156C	-989.2	-1067.3	Y156C	-1390.7	-1688.6
L159P	-998.8	-1144.4	L159P	-1176.4	-1392.8
R169C	-956.9	-1012.7	R169C	-1414.4	-1480
G183E	-801.1	-927.9	G183E	-1538.1	-1649.3
H196P	-865.7	-950.8	H196P	-1307.2	-1457.4
R240C	-994.2	-1043.6	R240C	-1444.6	-1499.8
R280P	-793.5	-861.5	R280P	-1513.7	-1513.7
F295S	-968.4	-1107.8	F295S	-1491.3	-1595.6
R296P	-870.8	-1082.9	R296P	-1460.9	-1492.2
R296W	-787.5	-925.8			

## Discussion

**Fig 4:**
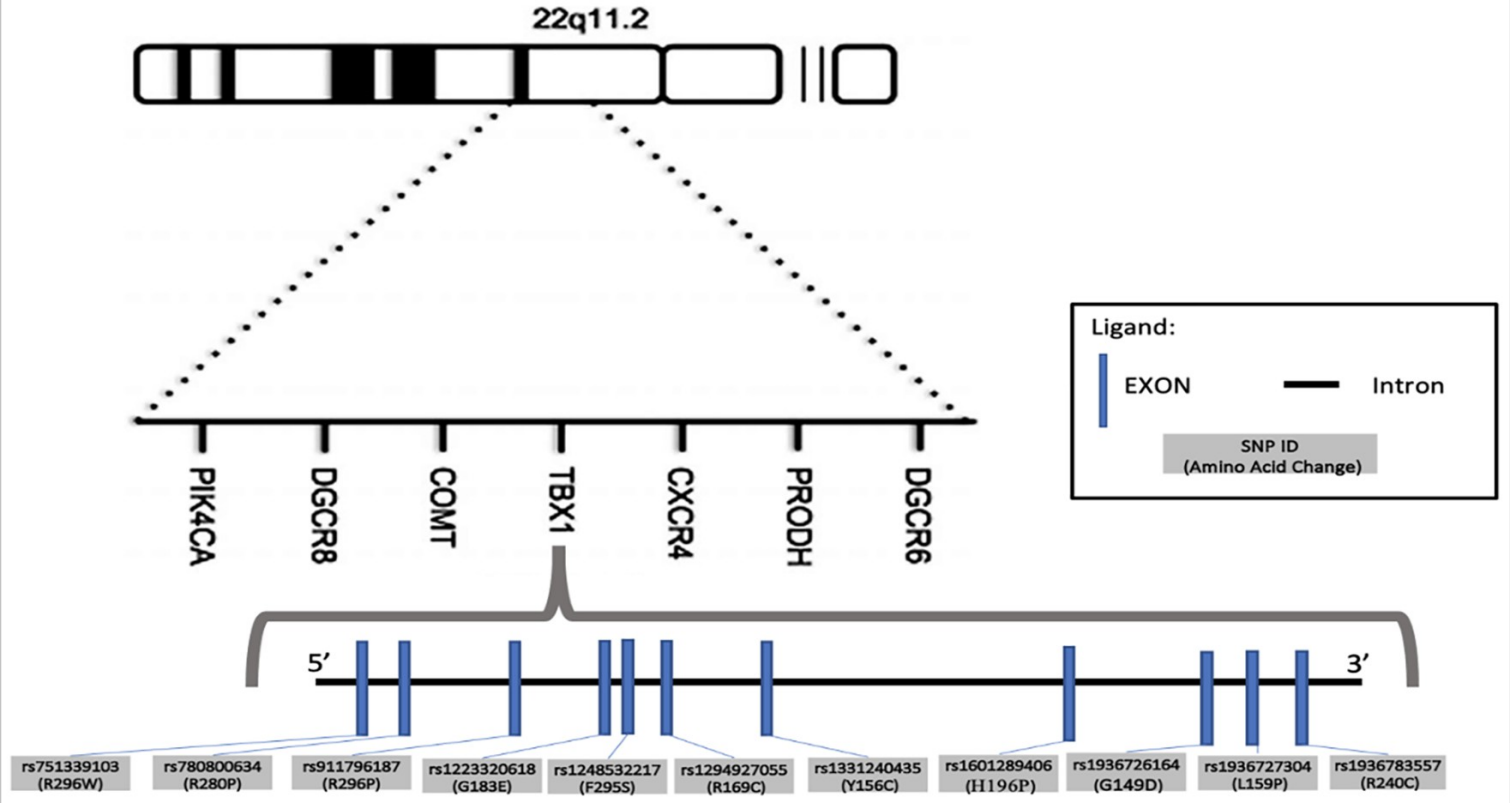
The eleven high-risk nsSNPs of *TBX1* identified in this study.

This study effectively employed in-silico analysis to identify eleven high-risk nsSNPs in the TBX1 gene: rs751339103 (C>A), rs780800634 (G>A), rs1936727304 (T>C), rs1223320618 (G>A), rs1248532217 (T>C), rs1294927055 (C>T), rs1331240435 (A>G), rs1601289406 (A>C), rs1936726164 (G>A), rs911796187 (G>A), and rs1331240435 (A>G) (Fig 4).

*TBX1* belongs to the T-box family of transcription factors, which are known for their critical role in various biological processes. Mutational studies have reported the occurrence of *TBX1* mutations in patients with 22q11.2DS features [36]. Identifying high-risk non-synonymous single-nucleotide polymorphisms (nsSNPs) in the *TBX1* gene using *in-silico* approaches can aid in diagnosing and treating 22q11.2DS. Early identification, diagnosis, and treatment are pivotal for managing 22q11.2DS effectively. Awareness of the patient’s long-term baseline condition and vigilantly monitoring shifts in emotions, cognition, sleep patterns, fatigue, physical health, behaviour, and overall functionality is important [37]. Understanding nsSNPS, researchers can unveil how these genetic changes influence disease development and progression. Thus, allowing customization of therapies or interventions that target the specific genetic anomalies associated with 22q11.2DS. This approach increases treatment efficacy and reduces adverse effects compared to a one-size-fits-all approach. This early intervention can potentially mitigate the severity of symptoms and improve long-term outcomes. Furthermore, Continuous monitoring of patients’ genetic profiles and their response to treatments can contribute to establishing prognosis models. It helps predict disease progression, identify potential complications, and refine treatment plans for better long-term care.

In this study, multiple computational tools were utilized to identify highly pathogenic variants, including PROVEAN, SIFT, PolyPhen-2, SNPs&GO, and PhD-SNP. The results from these tools predicted pathogenic or damaging nsSNPs, indicating their potential impact on protein function and stability. Among the analyzed variants, 70 nsSNPs were identified as pathogenic and was submitted to further analysis. Protein structure stability analysis indicated the rs1936727304 (T>C), rs911796187, rs1331240435, and rs1601289406 to cause alterations in interfaces, metal-binding sites, or allosteric sites, suggesting potential disruption of protein stability and function.

The impact of rs1936727304 (T>C), rs911796187, rs1331240435, and rs1601289406 on interfaces, metal-binding sites, or allosteric sites suggests a potential for disrupting protein stability and function. Integration of the results from protein stability analysis indicates alterations in the stability of the protein structure caused by these specific variations. Additionally, these variations affect protein-protein interactions crucial for proper functioning. Specifically, alterations at interfaces, metal-binding sites, or allosteric sites can compromise the protein’s structural integrity, changing how it interacts with other molecules or substrates and potentially affecting its function within cellular pathways or biological processes. These changes could render the protein more prone to misfolding, aggregation, or degradation, thereby impacting its overall stability and functionality. Furthermore, disruptions in protein-protein interactions resulting from these variations might interfere with crucial signalling cascades or molecular recognition events in the bone morphogenetic protein (BMP) pathway [3, 38].

Further analysis using PredictSNP, I-Mutant2.0, and MuPro tools validated the high-risk potential of 11 nsSNPs (Fig 4) and the R296P and Y156C were predicted to cause the loss of allosteric sites. In contrast, L159P and H196P were predicted to alter metal-binding sites. These conformational changes in internal dynamics could render the allosteric sites inaccessible for binding to these sequences [39].

Amino acid substitutions may affect hydrophobicity which destabilizes proteins by hindering stable conformation folding [40]. Research has shown that hydrophobic interactions contribute significantly (60 ± 4%) to protein stability [41]. In the current study, SWISS-MODEL generated 3D structures reveal that H196P, R280P, R296P, and R296W mutations introduce a more hydrophobic residue to *TBX1* than the wild-type residue, potentially reducing membrane interaction and protein flexibility. This results in decreased rigidity at that position. Conversely, G149D, Y156C, G183E, and F295S mutations introduced a more hydrophilic residue, likely reducing core hydrophobic interactions due to decreased hydrophobicity.

Furthermore, protein-protein interaction analysis highlighted the interaction of *TBX1* with various partner proteins such as *SEPT5*, *NKX2-5*, *BANF1*, *SLC25A1*, *GNB1L*, *UCP1*, *PRDM16*, *TMEM26*, *CIDEA*, and *DROSHA*. The study also identified associations between *TBX1* and the *FOX* gene family, particularly the *FOXC2* and *FOXC1* genes, suggesting their involvement in molecular pathways related to *TBX1*. The helix/forkhead box (Fox)-containing transcription factors crucial for regulating *TBX1* transcription in the pharyngeal endoderm and head mesenchyme [42, 43]. The Foxa2, vital for endoderm development [44, 45], along with Foxc1 or Foxc2, essential for head mesenchyme and aortic arch formation [46–48], bind and activate *TBX1* transcription within their respective subdomains through this regulatory element. Moreover, *TBX1* is required to express Foxa2 and Foxc2 in the pharyngeal endoderm and head mesenchyme. This intricate cascade of events culminates in the transcription of *TBX1*, representing the association of Foxc proteins during development [49].

Interaction between *TBX1* and signaling molecules like *FGF8* (Fibroblast Growth Factor 8) and *BMPER* (BMP-binding endothelial regulator) are pivotal in unravelling heart morphogenesis pathways. *FGF8* has been shown to play an essential role in heart development, specifically within Tbx1-expressing cells [50]. Similarly, *BMPER* play important role in modulating Bone Morphogenetic Protein (BMP) signaling, integral for skeletal and cardiovascular development [51]. This interaction signifies the regulatory role *TBX1* in modulating BMP signaling pathways, impacting crucial cardiac and skeletal development processes. Dysregulation in these interactions might underlie developmental disorders in DiGeorge syndrome, emphasizing the importance of comprehending these molecular pathways for potential targeted interventions. In this study, protein docking simulations between *TBX1* and *FGF8*/*BMPER* demonstrated the binding energies of different mutants and provided insights into the strength of interactions. Notably, the G149D and R296W in the *TBX1-FGF8* docking, and G183E and R296W in the *TBX1-BMPER* docking, exhibited lower energy scores, indicating stronger interactions between the proteins.

The comprehensive analysis of nsSNPs in the *TBX1* gene using various computational tools and methodologies has provided valuable insights into the potential pathogenicity, stability, functional impact, protein-protein interactions, and structural alterations caused by these variants. These findings contribute to our understanding of the molecular mechanisms underlying *TBX1*-related diseases and highlight the importance of further research to elucidate the precise role of these nsSNPs in disease development.

## Material and method

### nsSNPs extraction

The SNPs for *the TBX1* gene (Gene ID: 6899) were extracted from the NCBI dbSNP database, (https://www.ncbi.nlm.nih.gov/snp; accesses on April 2022). The protein sequences in FASTA format were retrieved from the UniProt (https://www.uniprot.org/uniprot). Non-synonymous SNPs (nsSNPs) were filtered and selected for analysis using multiple bioinformatic tools to detect highly pathogenic variants.

### Detection of deleterious and damaging nsSNPs

Potentially deleterious or damaging non-synonymous SNPs (nsSNPs) in the *TBX1* gene, were identified using five different bioinformatic tools: PROVEAN (http://provean.jcvi.org/), SIFT, PolyPhen-2 (http://genetics.bwh.harvard.edu/pph2/), PhD-SNP, and SNPs&GO (https://snps.biofold.org/snps-and-go/snps-and-go.html).

### Protein structure stability analysis

The impact of non-synonymous SNPs (nsSNPs) on protein structure stability, was assessed employing four different tools: PredictSNP (https://loschmidt.chemi.muni.cz/predictsnp1/, accessed in May 2022), I-Mutant2.0 (https://folding.biofold.org/cgi-bin/i-mutant2.0.cgi, accessed in June 2022), MUPro (https://mupro.proteomics.ics.uci.edu/, accessed in October 2022) and MutPred2 (http://mutpred.mutdb.org/, accessed on May 2022). These tools were utilized to predict the stability changes caused by the nsSNPs in the protein structure. The website predicts the effect of the amino acid substitution on the stability of the protein as well as the free energy value (DDG value) and the reliability index (RI) value of the amino acids. The RI value reveals the reliability of the prediction, where 0 indicates least reliable while a 10 indicates a most reliable result. The DDG value measures the energy changes between a folded and unfolded structure. When the DDG value is higher than zero, the mutation is said to be able to increase the protein stability whereas a negative DDG value will decrease the stability of the protein.

### Align-GVGD Biophysical Characteristic Analysis

The 70 nsSNPs predicted to be highly pathogenic, were further analysed for biophysical characteristic analysis using Align-GVGD (http://agvgd.hci.utah.edu/about.php, accessed in October 2022). This analysis aimed to predict the transactivation activity of each missense substitution in the *TBX1* gene.

### Observation of protein-protein interactions and molecular interaction networks

Protein-protein interactions in the context of the *TBX1* gene were analysed using STRING database (https://string-db.org/, accessed in December 2022), which provides a comprehensive collection of both observed and predicted protein-protein interactions. We visualized molecular interaction networks and biological pathways using the open source software platform Cytoscape (https://cytoscape.org/index.html, accessed in December 2022), which enables the integration of annotations, gene expression profiles, and other state data into these networks. Cytoscape was developed specifically for biological research and is a standard platform for complex network analysis and visualization.

### Prediction of structural alteration using SWISS-Mode

Structural alterations in the *TBX1* gene domain were determined using SWISS-Model (https://swissmodel.expasy.org/, accessed in October 2022), a web server that provides a fully automated server for protein homology modeling. There are three possible simulation modes for this task: computer mode, alignment mode, and project mode, depending on the level of user engagement. These models use conservative homology replacement procedures to model biologically relevant ligands and cofactors. The target sequence for the target protein must be in FASTA format, and SWISS-Model provides and develops a 3D structure that can be built in. We employed this tool to generate 3D models and predict the structural alterations in the *TBX1* gene domain.

### *TBX1* protein molecular docking

I-TASSER (Iterative Threading ASSEmbly Refinement) server was used to predict the structure of *TBX1*. Molecular docking was conducted utilising two web servers; pyDockWEB and ClusPro2.0. pyDockWEB is a web-based server that performs protein-protein docking using a scoring function based on interface residue pairs and energy potentials. It generates putative binding modes between *TBX1* and FGF8 or BMPER. ClusPro2.0 is another web-based server used for protein-protein docking. It employs a two-step approach involving global and local rigid-body docking to predict the binding poses between *TBX1* and FGF8 or BMPER. Binding affinity was determined to provide insights into the strength of the interactions between *TBX1* and *FGF8* or *BMPER*, indicating the likelihood of their binding.

## Conclusion

In conclusion, the *TBX1* protein plays a vital role in crucial function in the construction of the pharyngeal apparatus, the development of the heart, and tooth morphogenesis. This study reports 11 major nsSNPs in *TBX1*: rs1936727304 (T>C), rs911796187, rs1331240435, rs1601289406, rs1223320618 (G>A), rs751339103(C>A), rs780800634 (G>A), rs1294927055 (C>T), rs1936726164, and rs1248532217 (T>C) as high-risk nsSNPs. However, in vitro research is required to examine the effect of the protein’s structure and functionality in these mutations.

## Supporting information

S1 File Pathogenic *TBX1* nsSNPs. (DOCX)
