# Supplementary material for: In-silico identification of deleterious non-synonymous SNPs of TBX1 gene: Functional and structural impact towards 22q11.2DS
Source: PLoS One. 2024 Jun 21;19(6):e0298092. doi: 10.1371/journal.pone.0298092 (PMC11192383; doi:10.1371/journal.pone.0298092)
Supplement: S1 File — (DOCX) [file pone.0298092.s001.docx]

**Supplementary Data**

S1 Table: Pathogenic *TBX1* nsSNPs

| **SNP ID** |  |  | **PROVEAN** | **SIFT** | **PolyPhen-2** | **SNPs&GO** | **PhD-SNP** |
| --- | --- | --- | --- | --- | --- | --- | --- |
|  |  |  | **Pred** | **Pred** | **Effect** | **Pred** | **Pred** |
| rs41298814 | F140L | NP_542377.1 | Deleterious | Damaging | probably damaging | Disease | Disease |
| rs74315522 | H194Q | NP_542377.1 | Deleterious | Damaging | probably damaging | Disease | Disease |
| rs371125236 | D166N | NP_542377.1 | Deleterious | Damaging | probably damaging | Disease | Disease |
| rs746384751 | R171W | NP_542377.1 | Deleterious | Damaging | probably damaging | Disease | Disease |
| rs751339103 | R296W | NP_542377.1 | Deleterious | Damaging | probably damaging | Disease | Disease |
| rs761127806 | G127D | NP_542377.1 | Deleterious | Damaging | probably damaging | Disease | Disease |
| rs761127806 | G127A | NP_542377.1 | Deleterious | Damaging | probably damaging | Disease | Disease |
| rs766608075 | P197L | NP_542377.1 | Deleterious | Damaging | probably damaging | Disease | Disease |
| rs780800634 | R280P | NP_542377.1 | Deleterious | Damaging | probably damaging | Disease | Disease |
| rs1224140312 | M207V | NP_542377.1 | Deleterious | Damaging | probably damaging | Disease | Disease |
| rs1445910672 | E129K | NP_542377.1 | Deleterious | Damaging | probably damaging | Disease | Disease |
| rs1936725102 | P141S | NP_542377.1 | Deleterious | Damaging | probably damaging | Disease | Disease |
| rs1936727304 | L159P | NP_542377.1 | Deleterious | Damaging | probably damaging | Disease | Disease |
| rs1936812731 | A292G | NP_542377.1 | Deleterious | Damaging | probably damaging | Disease | Disease |
| rs546513284 | F213L | NP_542377.1 | Deleterious | Damaging | probably damaging | Disease | Disease |
| rs752484290 | A272V | NP_542377.1 | Deleterious | Damaging | probably damaging | Disease | Disease |
| rs754446709 | L147P | NP_542377.1 | Deleterious | Damaging | probably damaging | Disease | Disease |
| rs754818295 | R169H | NP_542377.1 | Deleterious | Damaging | probably damaging | Disease | Disease |
| rs760098496 | P489R | XP_016884414.1 | Deleterious | Damaging | probably damaging | Disease | Disease |
| rs760195570 | N221K | NP_542377.1 | Deleterious | Damaging | probably damaging | Disease | Disease |
| rs763559631 | F265L | NP_542377.1 | Deleterious | Damaging | probably damaging | Disease | Disease |
| rs766205381 | P164Q | NP_542377.1 | Deleterious | Damaging | probably damaging | Disease | Disease |
| rs766205381 | P164L | NP_542377.1 | Deleterious | Damaging | probably damaging | Disease | Disease |
| rs766405525 | R138Q | NP_542377.1 | Deleterious | Damaging | probably damaging | Disease | Disease |
| rs770313856 | R171Q | NP_542377.1 | Deleterious | Damaging | probably damaging | Disease | Disease |
| rs770380653 | Q277K | NP_542377.1 | Deleterious | Damaging | probably damaging | Disease | Disease |
| rs773107514 | R320C | NP_542377.1 | Deleterious | Damaging | probably damaging | Disease | Disease |
| rs774388985 | A204V | NP_542377.1 | Deleterious | Damaging | probably damaging | Disease | Disease |
| rs775979935 | F265L | NP_542377.1 | Deleterious | Damaging | probably damaging | Disease | Disease |
| rs776751131 | I286T | NP_542377.1 | Deleterious | Damaging | probably damaging | Disease | Disease |
| rs777394492 | D167H | NP_542377.1 | Deleterious | Damaging | probably damaging | Disease | Disease |
| rs909268372 | P200A | NP_542377.1 | Deleterious | Damaging | probably damaging | Disease | Disease |
| rs909599095 | G149S | NP_542377.1 | Deleterious | Damaging | probably damaging | Disease | Disease |
| rs911796187 | R296Q | NP_542377.1 | Deleterious | Damaging | probably damaging | Disease | Disease |
| rs911796187 | R296P | NP_542377.1 | Deleterious | Damaging | probably damaging | Disease | Disease |
| rs972345580 | M139L | NP_542377.1 | Deleterious | Damaging | probably damaging | Disease | Disease |
| rs983470703 | R192H | NP_542377.1 | Deleterious | Damaging | probably damaging | Disease | Disease |
| rs1043397159 | M160T | NP_542377.1 | Deleterious | Damaging | probably damaging | Disease | Disease |
| rs1174388102 | D161N | NP_542377.1 | Deleterious | Damaging | PROBABLY DAMAGING | Disease | Disease |
| rs1176085112 | R308P | NP_542377.1 | Deleterious | Damaging | PROBABLY DAMAGING | Disease | Disease |
| rs1196891106 | K217E | NP_542377.1 | Deleterious | Damaging | PROBABLY DAMAGING | Disease | Disease |
| rs1218308239 | N124I | NP_542377.1 | Deleterious | Damaging | PROBABLY DAMAGING | Disease | Disease |
| rs1223320618 | G183E | NP_542377.1 | Deleterious | Damaging | PROBABLY DAMAGING | Disease | Disease |
| rs1248532217 | F295S | NP_542377.1 | Deleterious | Damaging | PROBABLY DAMAGING | Disease | Disease |
| rs1294894854 | G227S | NP_542377.1 | Deleterious | Damaging | PROBABLY DAMAGING | Disease | Disease |
| rs1294927055 | R169C | NP_542377.1 | Deleterious | Damaging | PROBABLY DAMAGING | Disease | Disease |
| rs1316409370 | H196D | NP_542377.1 | Deleterious | Damaging | PROBABLY DAMAGING | Disease | Disease |
| rs1331240435 | Y156C | NP_542377.1 | Deleterious | Damaging | PROBABLY DAMAGING | Disease | Disease |
| rs1375795775 | F270L | NP_542377.1 | Deleterious | Damaging | PROBABLY DAMAGING | Disease | Disease |
| rs1383559813 | R240H | NP_542377.1 | Deleterious | Damaging | PROBABLY DAMAGING | Disease | Disease |
| rs1383642230 | A135D | NP_542377.1 | Deleterious | Damaging | PROBABLY DAMAGING | Disease | Disease |
| rs1383642230 | A135V | NP_542377.1 | Deleterious | Damaging | PROBABLY DAMAGING | Disease | Disease |
| rs1405239508 | H196Q | NP_542377.1 | Deleterious | Damaging | PROBABLY DAMAGING | Disease | Disease |
| rs1417510372 | M139I | NP_542377.1 | Deleterious | Damaging | PROBABLY DAMAGING | Disease | Disease |
| rs1601283525 | F123V | NP_542377.1 | Deleterious | Damaging | PROBABLY DAMAGING | Disease | Disease |
| rs1601289315 | D186A | NP_542377.1 | Deleterious | Damaging | PROBABLY DAMAGING | Disease | Disease |
| rs1601289406 | H196P | NP_542377.1 | Deleterious | Damaging | PROBABLY DAMAGING | Disease | Disease |
| rs1601290785 | P239L | NP_542377.1 | Deleterious | Damaging | PROBABLY DAMAGING | Disease | Disease |
| rs1829035066 | P485L | XP_016884414.1 | Deleterious | Damaging | PROBABLY DAMAGING | Disease | Disease |
| rs1936726164 | G149D | NP_542377.1 | Deleterious | Damaging | PROBABLY DAMAGING | Disease | Disease |
| rs1936762069 | T219S | NP_542377.1 | Deleterious | Damaging | PROBABLY DAMAGING | Disease | Disease |
| rs1936783085 | R236S | NP_542377.1 | Deleterious | Damaging | PROBABLY DAMAGING | Disease | Disease |
| rs1936783168 | Y237C | NP_542377.1 | Deleterious | Damaging | PROBABLY DAMAGING | Disease | Disease |
| rs1936783557 | R240C | NP_542377.1 | Deleterious | Damaging | PROBABLY DAMAGING | Disease | Disease |
| rs1936785926 | F263L | NP_542377.1 | Deleterious | Damaging | PROBABLY DAMAGING | Disease | Disease |
| rs1936786800 | T268I | NP_542377.1 | Deleterious | Damaging | PROBABLY DAMAGING | Disease | Disease |
| rs1936812110 | K285Q | NP_542377.1 | Deleterious | Damaging | PROBABLY DAMAGING | Disease | Disease |
| rs1936812655 | F291L | NP_542377.1 | Deleterious | Damaging | PROBABLY DAMAGING | Disease | Disease |
| rs1936813482 | D297H | NP_542377.1 | Deleterious | Damaging | PROBABLY DAMAGING | Disease | Disease |
| rs1936862657 | R482W | XP_016884414.1 | Deleterious | Damaging | PROBABLY DAMAGING | Disease | Disease |


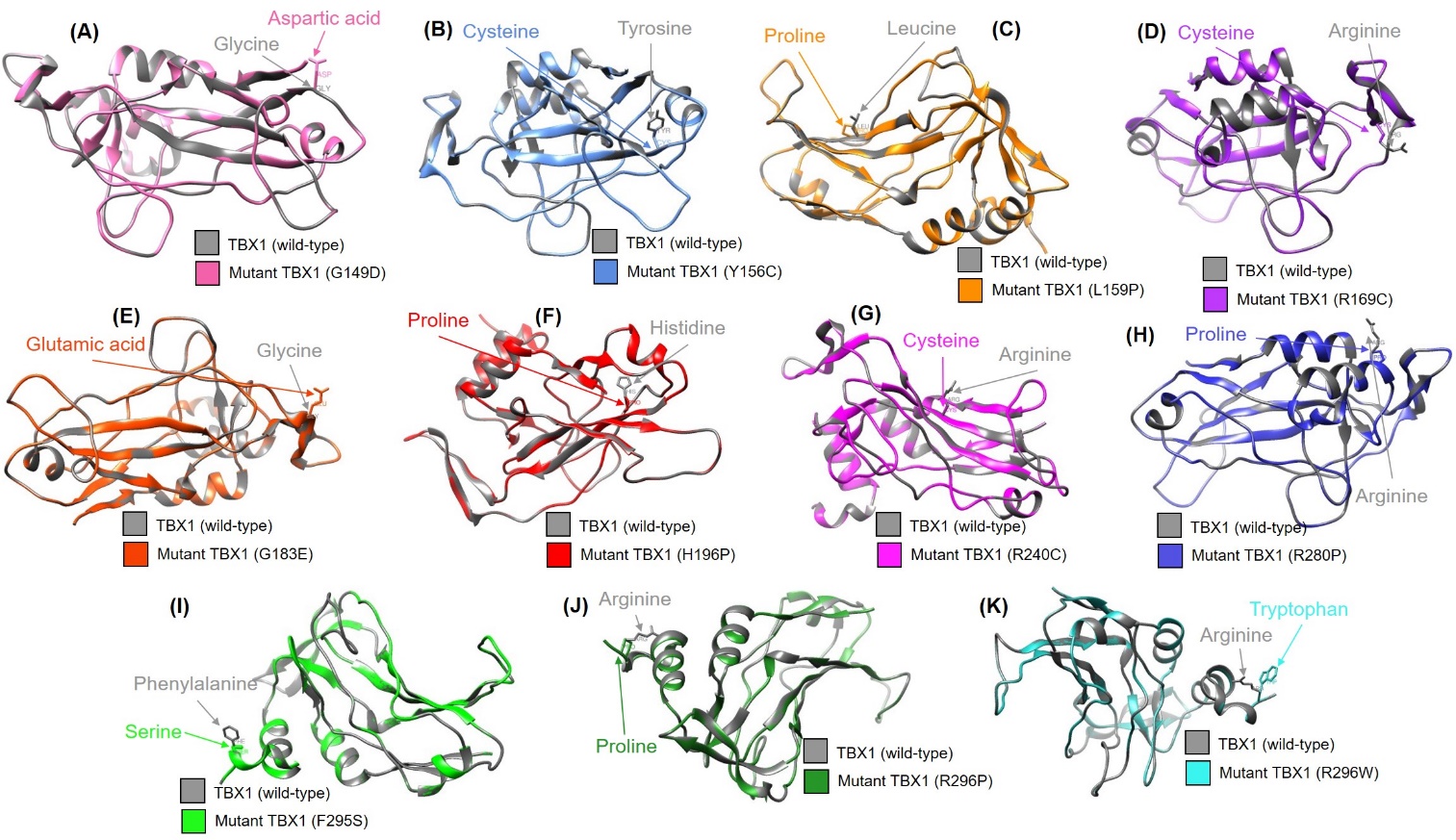


S2 Fig 1. Superimposed structures of the wild-type *TBX1* protein and the mutated proteins: (A) G149D, (B) Y156C, (C) L159P, (D) R169C, (E) G183E, (F) H196P, (G) R240C, (H) R280P, (I) F295S, (J) R296P, (K) R296W. 
